# Grade identification of ripened Pu-erh teas, and their differences of phenolic components, in vitro antioxidant capacity and hypoglycemic effect

**DOI:** 10.1016/j.fochx.2025.102421

**Published:** 2025-04-03

**Authors:** Cunqiang Ma, Bingsong Ma, Jiacai Wang, Zihao Wang, Binxing Zhou, Xuan Chen

**Affiliations:** a College of Horticulture, Nanjing Agricultural University, Nanjing 210095, China; b Qianxinan Academy of Agricultural and Forestry Sciences, Xingyi 562400, China; c Xinyang College of Agriculture and Forestry, Xinyang 464000, China; d College of Tea, Yunnan Agricultural University, Kunming 650201, China

**Keywords:** Dark tea, Inhibitory activity on *α*-amylase, Rutin, Theabrownins, Gallic acid, (−)-Epigallocatechin (PubChem Compound CID: 72277), (−)-Epicatechin (PubChem Compound CID: 72276), (−)-Epigallocatechin gallate (PubChem Compound CID: 65064), Gallic acid (PubChem Compound CID: 370), Rutin (PubChem Compound CID:5280805), Kaempferol (PubChem Compound CID: 5280863), Myricetin (PubChem Compound CID: 5281672), Quercetin (PubChem Compound CID: 5280343), Caffeine (PubChem Compound CID:2519).

## Abstract

Tea grade causes chemical differences. To reveal its detailed impact, chemical constitute and in vitro antioxidant capacity were determined in 20 ripened Pu-erh teas (RiPT). Their inhibitory activity on *α*-amylase and *α*-glucosidase were calculated to evaluate hypoglycemic effect. Results confirmed pile-fermentation as the main effective factor for chemical and functional differences among four series of RiPT. Furthermore, partial least squares-discriminant analysis and heat map analysis both accomplished the discrimination of high grade (G1) from middle grade (G3 and G5) and low grade (G7 and G9). Particularly, several phenolics like theaflavins, (−)-epigallocatechin (EGC), rutin and quercetin contributed to grade identification. Due to phenolics difference, RiPT grade showed positive correlation with antioxidant capacity and hypoglycemic effect. Characteristic antioxidants and inhibitors existed in RiPT with significantly positive (*P* < 0.05 and *r* > 0.75) correlations. Concretely, theaflavins, EGC, theabrownins, gallic acid, rutin and quercetin enhanced its antioxidant capacity and hypoglycemic effect.

## Highlights

• Pile-fermentation was major effective factor via its degree and microbial community structure.
• PLS-DA and heat map analysis distinguish high grade (G1) from middle and low grades.
• Phenolics like theaflavins, gallic acid, rutin, EGC with VIP > 1.0 for grade identification.
• Antioxidant capacity and hypoglycemic effect both show positive correlations to RiPT grade.
• Characteristic antioxidants and inhibitors such as theaflavins, quercetin, rutin exist in RiPT.

## Graphical abstract

**Figure:**
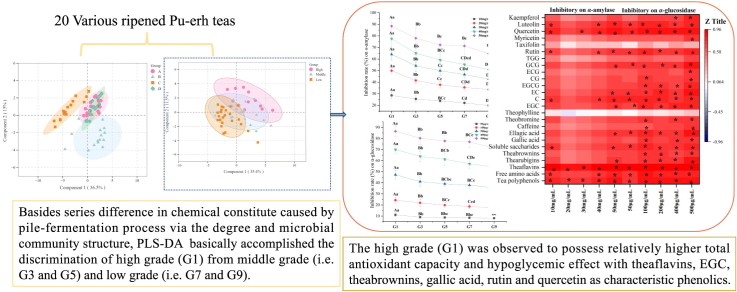

## 1 Introduction

Pu-erh teas, geographical indication product of China and having been on UNESCO's Representative List of the Intangible Cultural Heritage of Humanity as the subitem of ‘Chinese traditional tea-making skills and related customs’, are basically divided into two sub-types, i.e., raw Pu-erh tea (RaPT) and ripened Pu-erh tea (RiPT). Concretely, loose RiPT is produced from sun-dried green tea-leaves of (*Camellia sinensis* (Linn.) var. *assamica* (Masters) Kitamura) through pile-fermentation (spontaneous fermentation, or microbial fermentation), air drying, tea sieving and natural storage, for the production of RiPT cakes or bricks after blending, autoclave and second-drying (Ma et al., 2024). Based on the tenderness, aroma, taste and appearances by sensory evaluation, the loose RiPT are usually categorized into six grades, i.e., special grade, grade one (G1), grade three (G3), grade five (G5), grade seven (G7) and grade nine (G9). The comprehensive effect of raw material (e.g fresh tea-leaves) and processing technic such as enzymatic oxidation and microbial fermentation, resulted in chemical difference among various grades (Chen et al., 2024; Wang et al., 2021). Generally, green tea, yellow tea and white tea showed chemical consistency between tea grade and the tenderness of fresh tea-leaves, due to their non-fermentation or slight fermentation (Han et al., 2022). Comparatively, the pile-fermentation process enhances the grading difficulty. Scientific grading would help tea blending before the production of compressed RiPT. However, chemical and functional differences among various grades of RiPT have not been elucidated yet.

Like other dark teas such as Fu brick tea (Xiao et al., 2022), pile-fermentation is the critical process for flavor formation of RiPT, through extensive participation of various filamentous fungi (mould), yeast and bacteria (Zhao et al., 2019), which ensures its benefit superiority in anti-obesity, anti-diabetic and anti-cholesteremic activities, because of theabrownins accumulation (Huang et al., 2019; Wang, Qiu, Gan, & Zhu, 2022). Generally, during the pile-fermentation, catechins (flavanols), flavonol glycosides such as rutin, and free amino acids are dramatically decreased, while various catechin derivatives, tea polysaccharides and theabrownins are formed (Bian et al., 2022; Long et al., 2020; Ma et al., 2024). Besides the pile-fermentation degree showing positive correlation with the degradation rate of total polyphenols (Li et al., 2023), microbial community structure, particularly dominant fungi in the pile-fermentation, also profoundly impact RiPT chemical composition (Zhao, Su, et al., 2019). Nowadays, at least 93 fungi and 53 bacteria have been identified from the pile-fermentation (Ma et al., 2024). Thereinto, *Aspergillus niger, Aspergillus tubingensis, Aspergillus tamarii* and *Aspergillus fumigatus* enhance theabrownins content over 15.8 % of dry weight (DW) (Liao et al., 2023), while *Aspergillus pallidofulvus* and *Aspergillus sesamicola* promote the accumulations of gallic acid and several non-glycosylated flavonols such as quercetin and kaempferol (Wang et al., 2021; Ma et al., 2021). Different from the pile-fermentation dominated by *Aspergillus* genus, *Eurotium cristatum* contributes to flavor formation of Fu brick tea through the ‘flowering’ process (Xiao et al., 2022; Xiao et al., 2024), which indicates the leading role of dominant microbes in chemical variation during the microbial fermentation.

Despite several newly-invented technology, the traditional pile-fermentation with an output for about 2000 kg per batch still occupies the predominance for loose RiPT production (Ma et al., 2023). Due to the traditional pile-fermentation with a cycle over 30 days in an open environment, the single sample of each grade is difficult to summarize the variation rule of phenolic components and antioxidant capacity along with the grade of RiPT. Therefore, in this study, 20 RiPT samples of four series and five grades were collected to explore its change rule. In addition to quality components, phenolic compounds and purine alkaloids determined by spectrophotometer and high-performance liquid chromatography (HPLC), in vitro antioxidant capacity of various RiPT were evaluated by total antioxidant capacity (T-AOC), 1,1-diphenyl-2-picrylhydrazyl (DPPH) radical scavenging ability (DRSA), 2, 2′-azino-bis(3-ethylbenzothiazoline-6-sulfonic acid) (ABTS) radical scavenging ability (ARSA), hydroxyl radical scavenging ability (HRSA) and superoxide anion radical scavenging ability (SARSA), respectively. Furthermore, inhibitory ability on *α*-amylase and *α*-glucosidase enzymatic activity of serial concentrations were calculated to compare their in vitro hypoglycemic effect. This study elaborating specific impact of grade on chemical constitution, in vitro antioxidant capacity and hypoglycemic effect, would advance the knowledge about scientific grading and blending for the production of compressed RiPT.

## 2 Materials and methods

### 2.1 Materials and chemical reagents

According to evaluation criterion in “Product of geographical indication –Pu-erh tea” (GB/T 22111–2008), four series (i.e. A, B, C and D) of RiPT each with five grades (i.e. G1, G3, G5, G7 and G9) were collected in this work, which were all produced from sun-dried green tea-leaves of Menghai dayezhong (*Camellia sinensis* var. *assamica* cv. Mengku dayezhong), but were made at three different tea factories in Xishuangbanna Dai Autonomous Prefecture of Yunnan Province, China. Concretely, A and D series of RiPT were made at Menghai Tea Factory, while B series and C series were made at Yunchang Tea Factory and Fuding Tea Factory, respectively. The same series was produced from identical batch of raw material through traditional pile-fermentation and air drying in 2020 based on their processing experience. During natural storage after the pile-fermentation, RiPT grading was carried out by tea grade screening machine to obtain G1, G3, G5, G7 and G9, respectively from the same series of loose RiPT. Three biological duplication of each tea sample was executed, and these tea samples were maintained at −20 °C. The tea powder was collected through 40 mesh-filtration for chemical determinations, in vitro antioxidant capacity and in vitro hypoglycemic effect analysis.

Catechins standards including (+)-catechin (C, purity ≥99.8 %), epicatechin (EC, ≥ 99.4 %), (−)-epigallocatechin (EGC, ≥ 98.0 %), catechin gallate (CG, ≥ 98.0 %), (−)-epicatechin gallate (ECG, ≥ 98.0 %), (−)-gallocatechin gallate (GCG, ≥ 98.0 %) and (−)-epigallocatechin gallate (EGCG, ≥ 98.0 %), 1,3,6-tri-*O*-galloyl-*β*-d-glucose (TGG, a hydrolysable tannin ≥98.0 %), *α*-glucosidase (100 U), *p*-nitrophenyl-*α*-D-glucopyranoside (PNPG, ≥ 99.0 %) and acarbose (≥ 95.0 %) were purchased from Yuanye Bio-Technology Co., Ltd. (Shanghai, China). Four flavonols namely quercetin (≥ 98.0 %), kaempferol (≥ 98.0 %), myricetin (≥ 98.0 %) and rutin (quercetin-3-*O*-rutinoside, ≥ 98.0 %), taxifolin (a flavanonol, ≥ 98.0 %), luteolin (a flavone, ≥ 98.0 %), gallic acid (a phenolic acid, ≥ 98.0 %), ellagic acid (a phenolic acid, ≥ 98.0 %), and three purine alkaloids including caffeine (≥ 98.0 %), theobromine (≥ 99.0 %) and theophylline (≥ 98.0 %) were purchased from Must Bio-Technology Co., Ltd. (Chengdu, Sichuan, China). The *α*-amylase (5 U) was purchased from Sigma-Aldrich Co., Ltd. (St. Louis, MO, USA). Liquid chromatography-mass spectrometry (LC-MS) grade of methanol and acetonitrile were purchased from CNW Technologies GmbH Co., Ltd. (Bielefeld, North Rhine-Westphalia, Germany), and analytical reagent (AR) of ethyl alcohol, n-butyl alcohol, ethyl acetate and oxalic acid were purchased from Fengchuan Chemical Reagent Technology Co., Ltd. (Tianjin, China). Antioxidant capacity test kits of T-AOC, DRSA, ARSA, HRSA and SARSA were purchased from Geruisi Bio-Technology Co., Ltd. (Suzhou, Jiangsu, China).

### 2.2 Quality components determination

Moisture, water extracts, tea polyphenols and free amino acids contents were determined by the China National Institute of Standardization (CNIS) established methods of GB 5009.3–2016, GB/T 8305–2013, GB/T 8313–2018 and GB/T 8314–2013, respectively. Specifically, tea polyphenols content was measured by a UV-8000S ultraviolet-visible (UV–Vis) spectrophotometer (Yuanxi Instrument, Shanghai, China) at wavelength of 765 nm using Folin & Ciocalteu's phenol reagent with gallic acid (purity ≥98.5 %) regression curve, and the total content of free amino acids was determined by the UV–Vis spectrophotometer using ninhydrin assay with L-glutamic acid (≥ 99.0 %) regression curve. Additionally, the total content of soluble saccharides was measured by the UV–Vis spectrophotometer at 620 nm using D-(+)-glucose (≥ 99.0 %) regression curve (Ma et al., 2022; Wang et al., 2021). The total contents of theaflavins, thearubigins and theabrownins were systematically analyzed at 380 nm using the UV–Vis spectrophotometer (Liao et al., 2023; Ma et al., 2023).

### 2.3 HPLC determination of 16 phenolic compounds and 3 purine alkaloids

About 1000 mg of tea powder was extracted with 44 mL of methanol at 85 °C for 90 min, and then diluted to a volume of 50 mL by methanol (Ma et al., 2022). After centrifugation at 13,800 ×g (4 °C) for 10 min and filtration via a 0.45 μm nylon filter, 2 μL of tea extract was injected into an Agilent 1200 series HPLC system (Agilent Technologies, Santa Clara, CA, USA) comprised of Poroshell 120 EC-C_18_ chromatogram column (100 × 4.6 mm, 2.7 μm; Agilent Technologies, Santa Clara, CA, USA) and a Phenomenex C_18_ guard column (10 × 4.6 mm, 5 μm; Phenomenex, Torrance, CA, USA) for the determination of 16 phenolic compounds and 3 purine alkaloids according to the established method with slight modification (Ma et al., 2022; 2023). Solvent A (50 mL/L acetonitrile and 2.61 mL/L *ortho*-phosphoric acid solution) and solvent B (800 mL/L methanol solution) were prepared for HPLC separation with a stable flow rate of 0.8 mL/min and a column temperature of 30 °C. And their calibration curves with a high accuracy (R^2^ > 0.990) were established for the quantitative determination.

### 2.4 In vitro antioxidant capacity analysis by five various assays

#### 2.4.1 T-AOC

About 50 mg of RiPT powder was mixed with 1 mL of 80 % (*v/v*) ethanol-water solution for tea extract through ultrasonic extraction at 60 °C for 30 min. After centrifugation at 12,700 ×*g* for 10 min, their supernatant was determined by Multiskan FC series microplate reader (Thermo Scientific, Waltham, MA, USA) at 590 nm to calculate ferric ion reducing antioxidant power (FRAP) with Trolox as the standard.

#### 2.4.2 DRSA

About 50 mg of RiPT powder was mixed with 1 mL of 80 % (*v/v*) methanol-water solution for ultrasonic extraction (at 60 °C for 30 min). After centrifugation at 12,700 ×*g* for 10 min, their collected supernatant was determined by the microplate reader at 517 nm to calculate DRSA with Trolox as the standard.

#### 2.4.3 ARSA

About 50 mg of RiPT powder mixed with 1 mL of 80 % (*v/v*) methanol-water solution, was prepared for ultrasonic extraction at 60 °C with 30 min (shocking and mixing every 5 min). After centrifugation at 12,700 ×*g* for 10 min, their collected supernatant was determined by the microplate reader at 734 nm to calculate ARSA with Trolox as the standard.

#### 2.4.4 HRSA

About 100 mg of RiPT powder mixed with 1 mL of 80 % (*v/v*) ethanol-water solution was prepared for ultrasonic extraction at 50 °C with 10 min (shocking and mixing every 5 min). The supernatant was collected after centrifugation at 12,700 ×*g* for 10 min, and the absorbance at 510 nm was determined by the microplate reader to calculate the rate of HRSA referring to the kit procedure.

#### 2.4.5 SARSA

About 100 mg of RiPT powder was mixed with 1 mL of 80 % (*v/v*) ethanol-water solution for ultrasonic extraction at 50 °C with 30 min (shocking and mixing every 5 min). According to the kit procedure, the collected supernate after centrifugation at 12,700 ×*g* for 10 min was determined at 320 nm by the microplate reader to calculate the rate of SARSA.

### 2.5 In vitro hypoglycemic effect analysis

#### 2.5.1 Sample preparation of ripened Pu-erh tea extract (RiPTE)

About 25 g of tea powder mixed with 500 mL of distilled water was extracted at 95 °C for 30 min. After tea-leaf removal by absorbent carbasus, the filter liquor was collected through vacuum filtration using SHZ-IIID. The liquid supernatant was prepared through centrifugation at 6000 ×*g* for 20 min (25 °C). RiPTE powder was obtained by using HB 10 S96 Rotary Evaporator (IKA, Staufen, Germany) and FD5–3 Freeze Dryer (SIM International Group, Los Angeles, CA, USA). The determination assay about inhibitory rate of RiPTE on *α*-amylase and *α*-glucosidase enzyme activity followed previously described methods with some modifications (Guo, Long, Meng, Ho, & Zhang, 2018; Wen et al., 2023).

#### 2.5.2 Inhibitory ability analysis on *α*-amylase activity

The 500 μL of RiPTE solution with various concentrations (i.e. 10 mg/mL, 20 mg/mL, 30 mg/mL, 40 mg/mL and 50 mg/mL), or positive control (1 mM acarbose) was added into 500 μL of 13 U/mL *α*-amylase solution (in 20 mM sodium phosphate buffer with a pH of 6.9) and incubated in test tubes at 25 °C for 10 min. And then, 500 μL of 1 % (*w/v*) soluble starch solution that has been previously dissolved in sodium phosphate buffer and boiled for 15 min, was added into each tube and incubated for 25 min. After the addition of 1 mL of dinitrosalicylic acid color reagent, the tubes were placed in boiling water bath for 5 min to terminate enzyme reaction. Diluted with 100 mL of distilled water, the mixture was determined at 520 nm to obtain absorbance. The inhibition rate of RiPTE on *α*-amylase was calculated based on eq. (1).

(1) $$\text{Inhibition rate}\;\mathrm{on}\;\alpha -\text{amylase}\;\left(\%\right)=\left[A_{\mathrm{control}}-\left(A_{\mathrm{test}}-A_{\mathrm{blank}}\right)\right]/A_{\mathrm{control}}\times 100$$

In the eq. (1), *A*_*control*_ is the absorbance of sample without RiPTE, *A*_*test*_ is the absorbance of sample with RiPTE, and *A*_*blank*_ is the absorbance of sample with RiPTE, but without enzyme solution.

#### 2.5.3 Inhibitory ability analysis on *α*-glucosidase activity

In a 96-well plate, 50 μL of RiPTE solution with various concentrations (i.e. 50 μg/mL, 100 μg/mL, 200 μg/mL, 400 μg/mL, and 500 μg/mL), or positive control (1 mM acarbose) was added into 100 μL of 1 U/mL *α*-glucosidase solution (in 100 mM sodium phosphate buffer with a pH of 6.9) and incubated for 10 min. 50 μL of 5 mM PNPG solution in 0.1 mM sodium phosphate buffer (pH = 6.9) was added to each well and incubated 25 °C for 5 min. Their absorbance was read at 405 nm, and inhibition rate of RiPTE on *α*-glucosidase was calculated by eq. (2).

(2) $$\text{Inhibition rate}\;\mathrm{on}\;\alpha -\text{glucosidase}\;\left(\%\right)=\left[A_{\mathrm{control}}-\left(A_{\mathrm{test}}-A_{\mathrm{blank}}\right)\right]/A_{\mathrm{control}}\times 100$$

In the eq. (2), *A*_*control*_ is the absorbance of sample without RiPTE, *A*_*test*_ is the absorbance of sample with RiPTE, and *A*_*blank*_ is the absorbance of sample with RiPTE, but without enzyme solution.

### 2.6 Statistical analysis

Duplicate detection was executed to obtain reliable data represented as mean ± standard deviation. Partial least squares-discriminant analysis (PLS-DA) and principal component analysis (PCA) were performed using R package (version 3.5.1) at Metware cloud tool online platform (https://cloud.metware.cn). One-way analysis of variance (ANOVA) by Duncan's multiple range test and the bivariate correlation analysis were carried out using SPSS 20.0 for Windows (Armonk, NY, USA) to acquire significant difference levels and Person correlation coefficients, respectively. Origin 9.0 software (Hampton, Massachusetts, USA) was performed for heat map analysis and graphical exhibition.

## 3 Results and discussion

### 3.1 PLS-DA and chemical differences among four series of RiPT

At present, PCA has been developed for Pu-erh teas classification by processing technic (Ma et al., 2022), production region (Wang et al., 2018; Zhou et al., 2022) or storage period (Zhou et al., 2020). The same plant cultivar from Xishuangbanna region in this work provided relatively uniform raw material for the pile-fermentation of RiPT. However, the PCA could not simply divide these RiPT samples into series or grade (**Fig. S1**), which should be attributed to the synthetic effect of pile-fermentation process and tea grade. Overall, pile-fermentation process and tea grade together generated a total variance about 56.09 % in PCA model (PC1 = 39.68 % and PC2 = 16.41 %). Thereinto, the pile-fermentation was main effective factor for chemical compositions of RiPT. For instance, because of parameter difference in pile-fermentation process among various tea factories, the C series could be basically differentiated from other series by PCA.

**Fig. 1:**
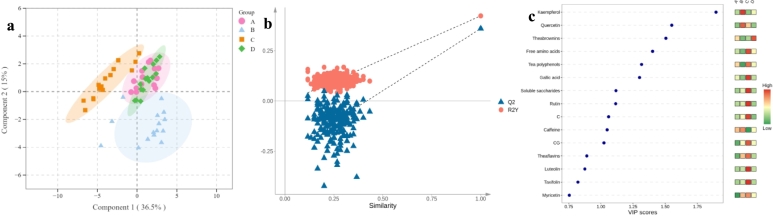
Statistical analysis of 25 chemical components for the series identification of RiPT. (**a**) Scores scatter plot of partial least -discriminant analysis (PLS-DA); (**b**) Permutation testing (200 times) in PLS-DA model; (**c**) Constitutional diagram of variable importance in the projection (VIP) value and heat map.

Other than the PCA, PLS-DA model (R^2^X = 0.515, R^2^Y = 0.423 and Q^2^ = 0.360, respectively) could basically accomplish the series identification with a certain acceptance (Fig. 1a and Fig. 1b). Except for partial reduplication, these RiPT samples were categorized as three groups, i.e., B group, C group, and A & D group, which was consistent with their manufacture factory. The same tea factory insured a high similarity of chemical constitute between A and D series. Conversely, the degree and microbial community structure of pile-fermentation resulted in chemical differences of RiPT among three tea factories. Therefore, the same tea factory could form its own characteristics due to similar degree and stable microbial community structure in the pile-fermentation. Among 25 detected chemicals, 3 flavonols (i.e. rutin, kaempferol and quercetin), 2 catechins (i.e. C and CG), tea polyphenols, theabrownins, soluble saccharides, free amino acids, gallic acid and caffeine with a variable importance in the projection (VIP) value >1.0 contributed to series discrimination and identification of RiPT through PLS-DA (Fig. 1c). The one-way ANOVA (**Table S1**) revealed significant (*P* < 0.05) or extremely significant (*P* < 0.001) differences of 6 quality components (i.e. tea polyphenols, free amino acids, soluble saccharides, theaflavins, thearubigins and theabrownins), 4 catechins (i.e. EGC, C, ECG and CG), 6 flavone/flavonols, 3 purine alkaloids and gallic acid among four series of RiPT. Particularly, the pile-fermentation caused free amino acids, thearubigins, gallic acid, theophylline, EGC, CG, ECG, rutin, taxifolin, myricetin, quercetin, luteolin and kaempferol with a maximum fold change (FC) > 1.50 (**Table S1**).

Through comparisons, the B series of RiPT had relatively higher pile-fermentation degree for its lower contents of tea polyphenols and free amino acids, while the C series showed lower pile-fermentation degree with significantly higher preservation of tea polyphenols, catechins and free amino acids, which was consistent with their dynamic changes during the pile-fermentation. Aerobic fermentation and anaerobic fermentation both promoted the mutual transformation of flavonols such as rutin, quercetin, myricetin and kaempferol through hydrolyzation, glycosylation, dehydroxylation, dehydroxylation and *O*-methylation under the catalyzation of relevant extracellular enzymes released by *Aspergillus*, *Blastobotrys* or lactic acid bacteria (Hou et al., 2023; Mao et al., 2024). Along with the degree enhancement, flavonol glycosides such as rutin also were highly transformed into relevant flavonols including quercetin, kaempferol and myricetin, through hydrolysis, dehydroxylation and hydroxylation. Generally, gallic acid and theabrownins were mainly originated from the hydrolysis of ester catechins, and the oxidative polymerization of catechins with amino acids and saccharides, respectively. However, the excessive pile-fermentation might restrain the continuous accumulation of theabrownins, even promote the degradation of gallic acid.

Besides the degree, the dominant fungi in microbial community structure of pile-fermentation, significantly impacted the contents of gallic acid, theabrownins, catechins, flavonols and related purine alkaloids. For instance, *A. tamarii, Aspergillus sydowii, Aspergillus ustus and Penicillium simplicissimum* have been confirmed to promote caffeine degradation or the mutual transformation of purine alkaloids (Yu et al., 2022; Zhou et al., 2020). Overall, the joint action of degree and microbial community structure in the pile-fermentation contributed to significant (*P* < 0.05) differences of gallic acid, theabrownins, the 4 catechins, 4 flavonols (i.e. rutin, quercetin, kaempferol and myricetin) and 3 purine alkaloids among four series of RiPT.

### 3.2 Comparison of in vitro antioxidant capacity and hypoglycemic effect among four series

Compared with RaPT, RiPT owned relatively lower in vitro antioxidant capacity for its lower level of phenolic components (Ma et al., 2022). However, the in vitro antioxidant capacity showed an obvious increase before the rapid decrease during the pile-fermentation. Especially, *A. pallidofulvus, A. tamarii* and *A. sesamicola* significantly (*P* < 0.05) enhanced in vitro antioxidant capacity in the microbial fermentation (Wang et al., 2021). The significant (*P* < 0.001) differences of T-AOC, DRSA, ARSA, HRSA and SARSA were found among four series of RiPT (**Table S2**). Generally, the pile-fermentation degree showed negative correlation to in vitro antioxidant capacity in RiPT. Therefore, the C series with the lowest degree contained the highest in vitro antioxidant capacity evaluated by T-AOC (365.7 ± 79.8 μmol Trolox/g), DRSA (129.1 ± 64.8 mg Trolox/g), ARSA (27.77 ± 10.92 mg Trolox/g) and HRSA (36.95 ± 2.38 %) among four series of RiPT. Only SARSA demonstrated a different trend from T-AOC, DRSA, ARSA and HRSA among the four series. Despite its relatively higher pile-fermentation degree, the B series had highest SARSA (52.21 ± 3.42 %), but lowest T-AOC, DRSA, ARSA and HRSA. In accordant with previous studies (Ma et al., 2022; Qie et al., 2021), T-AOC, DRSA, ARSA and HRSA, showed positive correlations with tea polyphenols, catechins, rutin and gallic acid contents. Therefore, chemical difference caused by pile-fermentation via its degree and microbial community structure, brought about extremely significant (*P* < 0.001) differences of in vitro antioxidant capacity among four series of RiPT.

The *α*-amylase and *α*-glucosidase are two primary enzymes involved in carbohydrate digestion. Inhibitory activities on *α*-amylase and *α*-glucosidase were used to evaluate in vitro hypoglycemic ability. In the present study, the inhibition abilities of RiPT on α-amylase and α-glucosidase enzymatic activity were determined to explore their difference in hypoglycemic effect among four series through one-way ANOVA (**Table S3**). Compared with *α*-amylase, the *α*-glucosidase showed higher sensibility to RiPTE for its lower concentrations. The inhibitory rate on α-glucosidase could reach over 80 % at a RiPTE concentration of 500 μg/mL. At all given RiPTE concentrations, inhibitory rates on *α*-amylase and *α*-glucosidase enzymatic activity both showed significant (*P* < 0.05) difference among the four series, which indicated the profound impact of pile-fermentation on in vitro hypoglycemic effect through its degree and microbial community structure. Generally, RiPT inhibition of *α*-amylase and *α*-glucosidase showed gradual decrease during the pile-fermentation (Long et al., 2020). By contrast, the C series with lower pile-fermentation degree was observed to possess highest inhibitory rate on *α*-amylase about 24.81 %, 41.49 %, 53.29 %, 67.39 % and 79.37 % at five RiPTE concentrations, respectively. However, the A series with a middle pile-fermentation degree contained an extremely significant (*P* < 0.001) higher inhibitory rate on *α*-glucosidase than other series at five given RiPTE concentrations. Concretely, their inhibitory rates on *α*-glucosidase were 9.95 %, 23.31 %, 44.44 %, 66.43 % and 81.99 %, respectively. Therefore, special microbial community in pile-fermentation might promote inhibition activity of *α*-amylase and *α*-glucosidase in RiPT, due to the generation of novel inhibitors such as bioactive peptides. At present, two bioactive peptides have been identified from RiPT with higher *α*-glucosidase inhibitory activity (Wang et al., 2024).

### 3.3 Grade identification of RiPT by PLS-DA

**Fig. 2:**
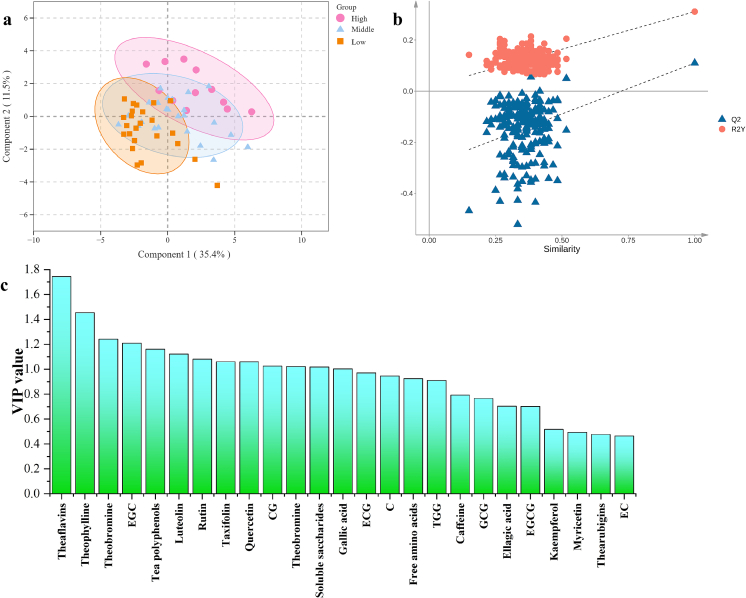
Partial least -discriminant analysis (PLS-DA) for grade identification of RiPT based on 25 chemical components. (**a**) Scores scatter plot of PLS-DA; (**b**) Permutation testing (200 times) in PLS-DA model; (**c**) Variable importance in the projection (VIP) value in PLS-DA.

During shoot growth of tea plant (*Camellia sinensis* L.), flavonoids such as catechins, and sugars were accumulated along with the decreases of amino acids and caffeine (Shen et al., 2019; Xiang et al., 2021). In accord with their distribution in fresh tea-leaves, the high-grade green teas such as Longjing tea and baked green tea contained relatively higher contents of amino acids and caffeine, but lower contents of catechins such as EGCG (Zou, Tang, Yang, Guo, & Song, 2023). Nevertheless, processing technic such as enzymatic oxidation of black tea, was able to alter chemical distribution of fresh tea-leaves among various grades (Chen et al., 2024; Guo et al., 2018; Ouyang et al., 2024). Based on 25 detected chemicals, including 6 quality components, 16 phenolic compounds and 3 purine alkaloids, PLS-DA (R^2^X = 0.469, R^2^Y = 0.311 and Q^2^ = 0.110, respectively) could basically accomplish the discrimination of high grade (i.e. G1) from the low grade (i.e. G7 and G9) (Fig. 2a). Additionally, the middle grade (i.e. G3 and G5) of RiPT fell between high grade and low grade in chemical constituent. Comparatively, the relatively lower Q^2^ in PLS-DA indicated that RiPT grade was secondary effective factor in chemical constituent, next to the pile-fermentation (Fig. 2b). Tea polyphenols, theaflavins, theabrownins, gallic acid, soluble saccharides, EGC, CG, taxifolin, rutin, quercetin and luteolin contributed to the grade identification of RiPT with a VIP value >1.0 (Fig. 2c), which mainly involved phenolic components such as tea pigments, catechins, phenolic acids and flavone/flavonols. Therefore, tea grading also led to distributional difference in phenolic components of RiPT, particularly theaflavins, EGC and theabrownins with a VIP value >1.20. Similar to the enzymatic oxidation in black tea production, the pile-fermentation completely transformed chemical distribution in raw material (sun-dried green tea-leaves) among various grades, due to their difference in microbial activity intensity.

### 3.4 Comparison of chemical differences among five grades of RiPT

**Fig. 3:**
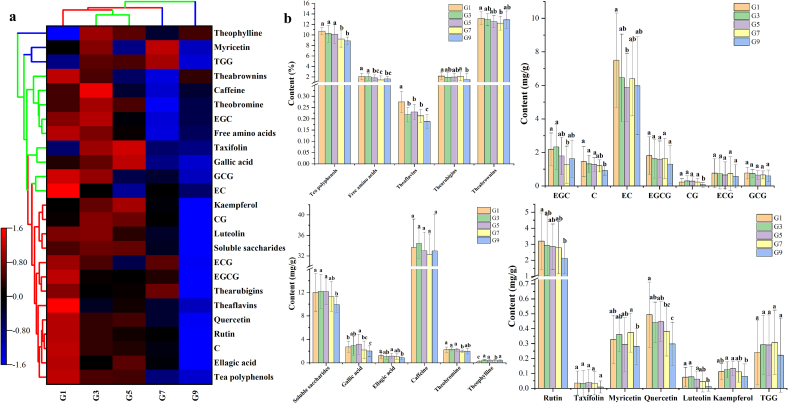
Phenolic components distribution and difference among five grades of RiPT through heat map analysis (**a**) and one-way ANOVA (**b**). Note: C, (+)-catechin; EC, (−)-epicatechin; EGC, (−)-epigallocatechin; ECG, (−)-epicatechin gallate; GCG, (−)-gallocatechin gallate; EGCG, (−)-epigallocatechin gallate; CG, (−)-catechin gallate; TGG, 1,3,6-tri-*O-*galloyl-β-d-glucose. Different lowercase letters above histogram (a, b and c, *P* < 0.05) indicate levels of statically significant difference determined by one-way ANOVA using Duncan's multiple range test.

Conformed with the PLS-DA result (Fig. 2), heat map analysis also divided these five grades into three groups, i.e., high (G1), middle (G3 and G5) and low (G7 and G9) group. Moreover, these 25 detected chemicals were divided into three clusters (Fig. 3a). Comparatively, the high grade of RiPT (G1) contained highest contents in cluster I or cluster II. Whereas the low grade such as G9 possessed lowest contents in cluster I and cluster II, but only with highest contents of mycetrin and TGG in cluster III. Generally, tea polyphenols, rutin, quercetin, EGCG and theaflavins in cluster I showed gradual decrease along with the grade decrease of RiPT. Additionally, gallic acid, EGC, theobromine and myricetin in cluster II showed an obvious increase after rapid decrease along with the grade decrease. As shown in Fig. 3b, only EGCG, ECG, GCG, caffeine, taxifolin and TGG kept relatively stable with no significant (*P* > 0.05) differences in RiPT among five grades. The 6 quality components (e.g. 3 tea pigments), 4 catechins (i.e. EGC, C, EC and CG), 5 flavones/flavonols (i.e. rutin, quercetin, kaempferol, myricetin and luteolin), 2 phenolic acids (i.e. gallic acid and ellagic acid) and 2 purine alkaloids (i.e. theobromine and theophylline) showed significant (*P* < 0.05) differences in RiPT among five grades.

Due to different intensity in microbial activity, the pile-fermentation process generated the consistent contents of ester catechins such as EGCG, ECG and GCG in RiPT among five grades, which was completely different from their distribution in green tea of various grades. Comparatively, the relatively higher intensity of microbial activity in the low grade of RiPT brought about profound reductions of ester catechins and tea polyphenols, while the high grade of RiPT (G1) had relatively higher reservations of free amino acids, tea polyphenols and soluble saccharides. Therefore, different from green tea and fresh tea-leaves, the pile-fermentation resulted in the realignment of phenolic components in RiPT among five grades. For instance, hydrolysable tannins such as digalloyl glucose and TGG for sweet aftertaste, showed positive correlations with the grade of green tea (Han et al., 2022). However, the pile-fermentation eliminated the difference of TGG content among five grades of RiPT through the hydrolyzation of hydrolysable tannins, decomposition of condensed tannins and subsequent oxidative polymerization. Additionally, Yunnan large leaf species and pile-fermentation process made for the higher caffeine content in RiPT over 32 mg/g of DW than that in other teas, which showed positive correlation with tea grade.

Apart from the hydrolyzation of ester catechins and hydrolysable tannins into relevant non-ester catechins and gallic acid, the joint action of moist-heat and microbial activity in the pile-fermentation further lead to the oxidation, condensation, degradation and polymerization of catechins and other phenolic compounds to formulate biological macromolecules such as theaflavins and theabrownins (Li et al., 2024). Theaflavins, thearubigins and theabrownins contents in high grade of RiPT (G1) were about 0.28 %, 2.17 % and 13.15 % of DW, respectively, for its dark brown formation of liquor color, which was significantly (*P* < 0.05) higher than that in middle and low grades. Previous studies (Hu et al., 2022; Ma et al., 2024) speculated that theabrownins were originated from the continuous oxidation of catechins with the polymerization of amino acids, flavonols and their glycosides such as quercetin, rutin, quercetin-3-*O-α*-L-rhamnopyranoside and kaempferol-3-*O-β*-D-glucopyranoside during the pile-fermentation. The higher theabrownins content in G1 should be attributed to its higher levels of free amino acids, rutin and quercetin with relatively higher reservation of catechins such as EC and C. Overall, the higher levels of free amino acids, theabrownins and flavonols in G1 elevated its umami and mellow taste, and health efficacy in anti-cholesterol (Huang et al., 2019). Furthermore, the high and middle grades of RiPT also contained significantly (*P* < 0.05) higher content of soluble saccharides than the low grade.

Compared with high and low grades, the middle grade of RiPT (i.e. G3 and G5) had relatively higher content of gallic acid, which should be attributable to massive hydrolysis of abundant ester catechins such as ECG and EGCG. Moreover, gallic acid could be transformed into ellagic acid through condensation during the pile-fermentation, which gave rise to the elevation of ellagic acid content in G1. As major flavonoids just second to catechins (flavanols) in content, various flavones/flavonols over 200 species have been identified in Pu-erh tea (Ma et al., 2024). Except for the stable taxifolin content, luteolin and the 4 flavonols showed significant (*P* < 0.05) differences in RiPT among five grades. Generally, the low grade such as G7 and G9 possessed significant (*P* < 0.05) lower contents of flavone/flavonols such as rutin, quercetin, luteolin and kaempferol. Through comparisons, the high grade of RiPT (G1) contained relatively higher contents of rutin, quercetin and luteolin, while the middle grade of RiPT possessed highest content of kaempferol and myricetin. Their higher contents of flavonols might improve RiPT health benefits such as delaying decline in global cognition (Holland et al., 2023).

### 3.5 Difference of in vitro antioxidant capacity in RiPT among five grades

**Fig. 4:**
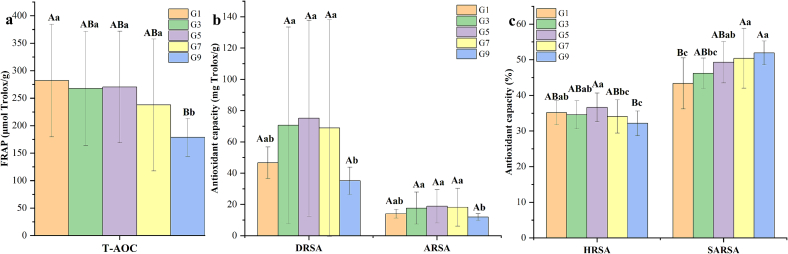
Difference of in vitro antioxidant capacity evaluated by five assays among five grades of RiPT. (**a**) Total antioxidant capacity (T-AOC) evaluated by ferric ion reducing antioxidant power (FRAP); (**b**) DPPH radical scavenging ability (DRSA) and ABTS radical scavenging ability (ARSA); (**c**) Hydroxyl radical scavenging ability (HRSA) and superoxide anion radical scavenging ability (SARSA). Note: Different uppercase and lowercase letters above histogram (A and B, *P* < 0.001; a, b and c, *P* < 0.05) indicated levels of statically significant difference determined by one-way ANOVA using Duncan's multiple range test.

Both enzymatic oxidation and microbial fermentation in tea processing promoted the reduction of in vitro antioxidant capacity along with the decrease of total polyphenols content. Generally, dark tea such as RiPT, Liubao tea and Fu brick tea, and black tea had relatively lower in vitro antioxidant capacity than non-fermented teas such as green tea and yellow tea (Zhao et al., 2019). Due to that some phenolic compounds such as catechins cannot pass the small intestinal barrier, the in vivo antioxidant activity was radically different from in vitro antioxidant capacity in teas (Ma et al., 2022). As shown in Fig. 4, other than the difference of in vitro antioxidant capacity among four series caused by pile-fermentation, significant (*P* < 0.05) differences of T-AOC, DRSA, ARSA, HRSA and SARSA also were found among five grades, indicating profound impact of RiPT grade on in vitro antioxidant capacity. The higher contents of phenolic components including catechins, flavonols and flavonol glycosides, were responsible for the highest T-AOC about 281.1 ± 102.6 μmol Trolox/g in the high grade of RiPT (G1). Comparatively, the middle grade of RiPT (i.e. G3 and G5) possessed significantly (*P* < 0.05) higher DRSA (about 70.63–75.13 mg Trolox/g), ARSA (about 17.65–18.79 mg Trolox/g) and HRSA (34.55–36.63 %) than other grades (**Table S4**). In RiPT, the in vitro antioxidant capacity such as T-AOC, DRSA, ARSA and HRSA showed positive correlation to tea grade. For instance, the low grade of RiPT such as G7 and G9 had lowest levels of T-AOC, DRSA, ARSA and HRSA, but with highest SARSA. Their antioxidants distribution in various grades brought about the difference of in vitro antioxidant capacity, which deserved further investigation through correlation analysis.

### 3.6 Comparison of inhibitory activity on α-amylase and α-glucosidase among five grades

**Fig. 5:**
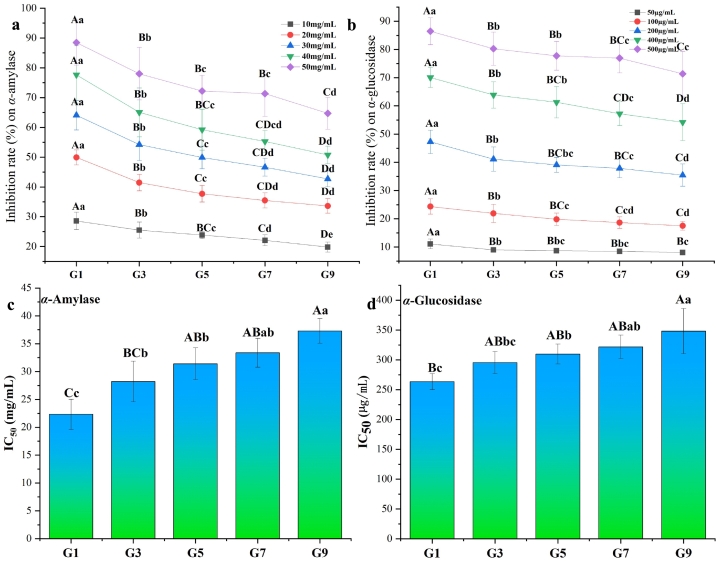
RiPTE inhibitory rates of five grades with five concentrations on *α*-amylase (**a**) and *α*-glucosidase (**b**), and their half inhibition concentration(IC_50_) values (**c** and **d**). Note: Different uppercase and lowercase letters above line (A, B, C and D, *P* < 0.001; a, b, c, d and e, *P* < 0.05) indicated levels of statically significant difference determined by one-way ANOVA using Duncan's multiple range test.

As a metabolic disease, diabetes mellitus is mainly caused by diminished secretion of insulin, or poor responses of organs to insulin. Postprandial hyperglycemia shows strong correlation with the development of diabetes (Zhu, Chen, Zhang, & Huang, 2020). Pu-erh tea has been confirmed with anti-hyperglycaemic effect to reduce postprandial hyperglycemia for its abundant polysaccharides, flavonoids, phenolic acids and bioactive peptides (Wang et al., 2024; Yang et al., 2019). However, the difference of hypoglycemic effect still remains unknown among various grades of RiPT. Like previous studies (Long et al., 2020; Wen et al., 2023), inhibitory activities of RiPT on *α*-amylase and *α*-glucosidase were usually developed to evaluate in vitro hypoglycemic effect. As shown in Fig. 5, the inhibitory rate on *α*-amylase and *α*-glucosidase could reach over 85 % at the high concentrations of PiPTE (e.g. 50 mg/mL and 500 μg/mL), which indicated that the RiPTE had similar hypoglycemic effect to positive control (acarbose). Additionally, the RiPTE concentration showed linear positive correlation with inhibitory rate on *α*-amylase and *α*-glucosidase.

The one-way ANOVA revealed extremely significant (*P* < 0.001) difference in inhibitory rate of RiPT on *α*-amylase and *α*-glucosidase among five grades (Fig. 5a and Fig. 5b). The high grade of RiPT (G1) was confirmed with highest inhibitory rate on *α*-amylase and *α*-glucosidase. Specifically, its inhibitory rates on *α*-amylase were about 64.08 %, 77.62 % and 88.47 % at 30 mg/mL, 40 mg/mL and 50 mg/mL of RiPTE, respectively. And, its inhibitory rates on *α*-glucosidase were about 47.27 %, 70.08 % and 86.44 % at a RiPTE concentration of 200 μg/mL, 400 μg/mL and 500 μg/mL, respectively. Generally, the RiPT grade showed positive correlation with the inhibitory rate on *α*-amylase and *α*-glucosidase. Similar decreasing trend of inhibitory activity along with grade decrease could be found in all series of RiPT.

At present, half inhibition concentration (IC_50_) values of green tea, black tea, white tea, oolong tea and pickled tea (Wen et al., 2023) on *α*-amylase and *α*-glucosidase have been calculated, respectively. Through comparisons, green tea had lowest IC_50_ values on *α*-amylase and *α*-glucosidase about 6.31 mg/mL and 90 μg/mL (Liu, Ai, Qu, Chen, & Ni, 2017). Correspondingly, the enzymatic oxidation enhanced the IC_50_ values of black tea on *α*-amylase and *α*-glucosidase. Similar to black tea, RiPT had IC_50_ value on *α*-amylase activity about 22.34–37.3 mg/mL, and IC_50_ value on *α*-glucosidase about 263.49–348.22 μg/mL. Apart from pile-fermentation, the RiPT grade also extremely significantly (*P* < 0.001) affected IC_50_ values (Fig. 5c and Fig. 5d). The IC_50_ value on *α*-amylase and *α*-glucosidase both gradually increased along with the grade increasing. Specifically, the high grade of RiPT (G1) had IC_50_ values about 22.34 ± 2.71 mg/mL on *α*-amylase, and about 263.49 ± 13.44 μg/mL on *α*-glucosidase, which were just about 60–75 % of their IC_50_ values in G7 RiPT. These results elucidated that RiPT grade was main effective factor of inhibitor activity on *α*-amylase and *α*-glucosidase. The phenolic components such as catechins, theaflavins and flavonols would be potential inhibitors in RiPT for higher hypoglycemic effect.

### 3.7 Correlation analysis to select marker, and potential antioxidants and inhibitors

In this work, correlation analysis was carried out to explore markers for grade identification, and potential antioxidants and inhibitors based on their Pearson correlation coefficients. Tea polyphenols, free amino acids, theaflavins, soluble saccharides, ellagic acid, theobromine, EGC, C, EGCG, EC, ECG, GCG, rutin, quercetin and luteolin contents in RiPT showed significantly negative (*P* < 0.05 and *r* < −0.75) correlation with the grade. Combined with their VIP values in PLS-DA and Pearson correlation coefficients in correlation analysis, tea polyphenols, theaflavins, rutin, quercetin and luteolin were regarded as markers of G1, for the identification of high grade. Furthermore, these markers also showed gradual decrease along with the grade decrease.

**Fig. 6:**
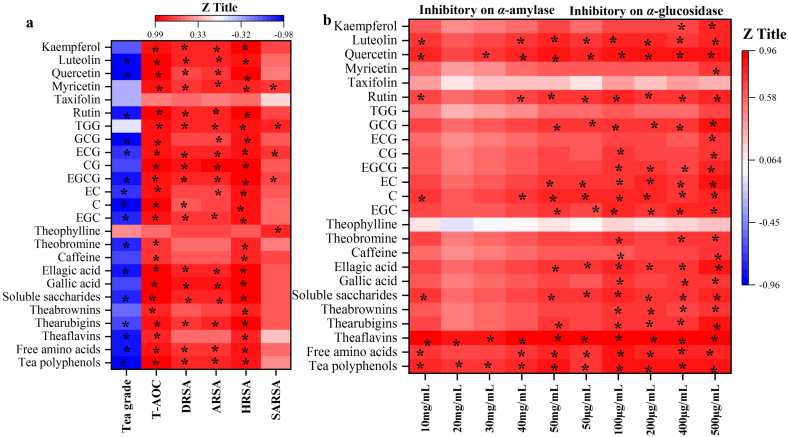
Heat map representing Pearson correlation coefficients of 25 detected chemicals to RiPT grade, in vitro antioxidant capacity (**a**) and hypoglycemic effect (**b**), respectively. Note: * indicated the significant correlation at *P* < 0.05 level.

Because of their significantly positive (*P* < 0.05 and *r* > 0.75) correlations, 7 catechins, 2 phenolic acids, TGG and 5 flavone/flavonols (i.e. rutin, myricetin, quercetin, luteolin and kaempferol) were major antioxidants for T-AOC in RiPT (Fig. 6a). As major water-soluble pigments in tea, theaflavins, thearubigins and theabrownins also were conducive to T-AOC, DRSA, ARSA and HRSA in RiPT with significantly positive (*P* < 0.05 and r > 0.75) correlations. Potential antioxidants for T-AOC, DRSA, ARSA, HRSA and SARSA were different in RiPT. For instance, thearubigins, gallic acid, ellagic acid, EGC, EGCG, CG, ECG, TGG, rutin, myricetin, luteolin and kaempferol were confirmed as potential antioxidants for T-AOC, DRSA, ARSA and HRSA in RiPT, while only ECG and TGG contributed to the in vitro antioxidant ability evaluated by SARSA. Particularly, rutin, quercetin, EC, theaflavins, theabrownins, gallic acid and EGCG led to relatively higher in vitro antioxidant capability of T-AOC, DRSA, ARSA or HRSA in high grade of RiPT. Overall, RiPT demonstrated differentiated antioxidant distribution from RaPT.

Inhibitory rates of various RiPTE concentrations on *α*-amylase and *α*-glucosidase were used for correlation analysis (Fig. 6b). Phenolic components including theaflavins, ellagic acid, EGC, EC, C, GCG, rutin, quercetin and luteolin, showed significantly positive (*P* < 0.05 and *r* > 0.75) correlations to inhibitory activity both on *α*-amylase and *α*-glucosidase. Furthermore, thearubigins, theabrownins, gallic acid, EGCG and kaempferol also showed significantly positive (*P* < 0.05 and r > 0.75) correlations to inhibitory activity on *α*-glucosidase. Overall, 5 catechins (i.e. EGC, EC, C, GCG and EGCG), 4 flavone/flavonols (i.e. rutin, quercetin, kaempferol and luteolin), 2 phenolic acids and 3 tea pigments were effective inhibitors in RiPT of *α*-amylase or *α*-glucosidase activities for the formation of stable and tight complexes through hydrogen bonding, hydrophobic interactions, and van der Waals forces. Because the mixture of rutin and quercetin promoted the pectin inhibition of *α*-amylase (Qin et al., 2025), rutin and quercetin might be major inhibitors in high grade of RiPT (G1) for its highest inhibitory activity. Additionally, theaflavins, EC, gallic acid, theabrownins and EGCG also enhanced RiPT inhibition activity of *α*-amylase or *α*-glucosidase. Therefore, the high grade of RiPT would be used as research material to further investigate inhibitors of hypoglycemic effect through modern medicine and LC-MS technology.

## 4 Conclusion

Phenolic compounds and in vitro antioxidant capacity in 20 RiPT samples of four series and five grades were comprehensively investigated by HPLC and five assay methods, respectively. And their hypoglycemic effect were assessed by inhibitory ability on *α*-amylase and *α*-glucosidase activity. Although the degree and microbial community structure in pile-fermentation generated significant (*P* < 0.05) differences of chemical constitute, in vitro antioxidant capacity and hypoglycemic effect among four series of RiPT, PLS-DA and heat map analysis both confirmed that these RiPT could be basically divided into high grade (G1), middle grade (i.e. G3 and G5) and low grade (i.e. G7 and G9). Thereinto, theaflavins, EGC, rutin, quercetin, luteolin, gallic acid, taxifolin and CG with a VIP value >1.0 were beneficial for grade identification. Particularly, theaflavins, rutin and quercetin were markers in the high grade (G1). Through comparisons, the high grade of RiPT was observed to possess significantly (*P* < 0.05) higher T-AOC, and inhibitory activity on *α*-amylase with IC_50_ about 22.34 mg/mL and *α*-glucosidase with IC_50_ about 263.49 μg/mL than other grades. Due to their significantly positive (*P* < 0.05 and r > 0.75) correlations, correlation analysis confirmed rutin, quercetin, EC, theaflavins and EGCG as antioxidants in RiPT for higher in vitro antioxidant capacity. Additionally, rutin, quercetin, theaflavins, EC, gallic acid, theabrownins and EGCG as potential inhibitors led to the highest hypoglycemic effect in the high grade. This study revealed chemical and health efficacy difference among five RiPT grade, which would provide theoretical foundation for tea blending in RiPT production.

## CRediT authorship contribution statement

**Cunqiang Ma:** Writing – review & editing, Writing – original draft, Visualization, Formal analysis, Conceptualization. **Bingsong Ma:** Validation, Resources. **Jiacai Wang:** Methodology, Data curation. **Zihao Wang:** Funding acquisition, Conceptualization. **Binxing Zhou:** Software, Methodology, Funding acquisition. **Xuan Chen:** Writing – review & editing, Supervision, Project administration.

## Ethics approval

Not applicable.

## Declaration of competing interest

The authors declare that they have no known competing financial interests or personal relationships that could have appeared to influence the work reported in this paper.

## Data Availability

Data will be made available on request.

## Appendix A Supplementary data

Supplementary material **Table S1.** Differences of 6 major quality components, 16 phenolic compounds and 3 purine alkaloids in ripened Pu-erh tea (RiPT) among four series. **Table S2.** Differences of in vitro antioxidant capacity in ripened Pu-erh tea (RiPT) among four series. **Table S3**. Inhibitory activities of various concentrations of ripened Pu-erh tea extract (RiPTE) on *α*-amylase and *α*-glucosidase, and their differences among four series. **Table S4.** Differences of in vitro antioxidant capacity in ripened Pu-erh tea (RiPT) among five grades. **Fig. S1.** Principal component analysis (PCA) of 20 ripened Pu-erh tea (RiPT) samples for classification of series (a) and grade (b), respectively. Supplementary material
