# Supplementary material for: Grade identification of ripened Pu-erh teas, and their differences of phenolic components, in vitro antioxidant capacity and hypoglycemic effect
Source: Food Chem X. 2025 Apr 3;27:102421. doi: 10.1016/j.fochx.2025.102421 (PMC12005310; doi:10.1016/j.fochx.2025.102421)
Supplement: Supplementary material — Table S1. Differences of 6 major quality components, 16 phenolic compounds and 3 purine alkaloids in ripened Pu-erh tea (RiPT) among four series. Table S2. Differences of in vitro antioxidant capacity in ripened Pu-erh tea (RiPT) among four series. Table S3. Inhibitory activities of various concentrations of ripened Pu-erh tea extract (RiPTE) on α-amylase and α-glucosidase, and their differences among four series. Table S4. Differences of in vitro antioxidant capacity in ripened Pu-erh tea (RiPT) among five grades. Fig. S1. Principal component analysis (PCA) of 20 ripened Pu-erh tea (RiPT) samples for classification of series (a) and grade (b), respectively. [file mmc1.docx]

**Table S1.** Differences of 6 major quality components, 16 phenolic compounds and 3 purine alkaloids in ripened Pu-erh tea (RiPT) among four series.

| Index | A (n=5×3×2) | B (n=5×3×2) | C (n=5×3×2) | D (n=5×3×2) | F-value |
| --- | --- | --- | --- | --- | --- |
| Tea polyphenols (%) | 10.20±1.28^ABb^ | 8.27±1.39^Cc^ | 11.12±1.05^Aa^ | 9.72±0.49^Bb^ | 34.82 |
| Free amino acids(%) | 1.55±0.38^BCc^ | 1.21±0.51^Cd^ | 2.59±0.44^Aa^ | 1.83±0.34^Bb^ | 58.21 |
| Theaflavins(%) | 0.205±0.028^Bc^ | 0.234±0.063^ABab^ | 0.245±0.041^Aa^ | 0.215±0.028^ABbc^ | 5.42 |
| Thearubigins(%) | 2.06±0.96^ABab^ | 1.70±0.67^Bbc^ | 2.45±0.83^Aa^ | 1.56±0.75^Bc^ | 7.14 |
| Theabrownins(%) | 12.99±1.69^Bb^ | 11.79±0.75^Cc^ | 12.16±0.58^BCc^ | 13.97±0.80^Aa^ | 25.57 |
| Soluble saccharides(mg/g) | 10.18±2.18^Bb^ | 11.18±2.30^Bb^ | 14.33±2.33^Aa^ | 10.37±1.12^Bb^ | 26.57 |
| Gallic acid(mg/g) | 2.42±1.51^Bb^ | 1.54±0.64^Cc^ | 4.46±0.70^Aa^ | 2.00±0.34^BCbc^ | 60.82 |
| Ellagic acid(mg/g) | 1.07±0.46^Aa^ | 0.99±0.46^Aa^ | 1.01±0.47^Aa^ | 1.18±0.52^Aa^ | 0.99 |
| Caffeine(mg/g) | 34.86±5.01^Aab^ | 36.04±5.52^Aa^ | 29.02±2.98^Bc^ | 33.21±1.76^Ab^ | 16.78 |
| Theobromine(mg/g) | 2.19±0.51^Aab^ | 2.35±0.39^Aa^ | 2.14±0.27^Aab^ | 2.05±0.10^Ab^ | 3.90 |
| Theophylline(mg/g) | 0.242±0.252^Bb^ | 0.426±0.094^Aa^ | 0.367±0.191^ABa^ | 0.385±0.063^ABa^ | 6.71 |
| EGC(mg/g) | 1.99±1.59^ABa^ | 2.04±0.76^ABa^ | 1.10±1.31^Bb^ | 2.22±0.25^Aa^ | 6.15 |
| C(mg/g) | 1.07±0.44^Bbc^ | 1.24±0.75^ABb^ | 1.65±0.39^Aa^ | 0.97±0.25^Bc^ | 11.19 |
| EC(mg/g) | 6.35±2.43^Aa^ | 6.52±3.06^Aa^ | 7.02±2.65^Aa^ | 5.86±1.98^Aa^ | 1.06 |
| EGCG(mg/g) | 1.37±0.96^Aa^ | 1.66±1.23^Aa^ | 1.88±1.16^Aa^ | 1.45±1.15^Aa^ | 1.24 |
| CG(mg/g) | 0.011±0.059^Cc^ | 0.216±0.155^Bb^ | 0.449±0.293^Aa^ | 0.206±0.192^Bb^ | 25.05 |
| ECG(mg/g) | 0.574±0.633^Ab^ | 0.667±0.750^Aab^ | 1.108±1.215^Aa^ | 0.406±0.467^Ab^ | 4.06 |
| GCG(mg/g) | 0.692±0.243^Aa^ | 0.646±0.266^Aa^ | 0.750±0.357^Aa^ | 0.638±0.303^Aa^ | 0.91 |
| TGG(mg/g) | 0.287±0.224^Aa^ | 0.295±0.194^Aa^ | 0.258±0.194^Aa^ | 0.242±0.251^Aa^ | 0.39 |
| Rutin(mg/g) | 2.16±0.84^Bb^ | 2.48±1.53^Bb^ | 4.47±1.29^Aa^ | 1.95±1.16^Bb^ | 26.65 |
| Taxifolin(mg/g) | 0.067±0.037^Bb^ | 0.019±0.059^ABb^ | 0.081±0.110^Aa^ | 0.011±0.059^Bb^ | 7.11 |
| Myricetin(mg/g) | 0.249±0.155^Ab^ | 0.357±0.141^Aa^ | 0.376±0.152^Aa^ | 0.325±0.104^Aa^ | 4.81 |
| Quercetin(mg/g) | 0.331±0.143^Bc^ | 0.532±0.188^Aa^ | 0.449±0.135^ABb^ | 0.335±0.146^Bc^ | 11.88 |
| Luteolin(mg/g) | 0.034±0.064^Bb^ | 0.040±0.056^Bb^ | 0.105±0.066^Aa^ | 0.039±0.039^Bb^ | 10.25 |
| Kaempferol(mg/g) | 0.084±0.047^Bbc^ | 0.186±0.053^Aa^ | 0.070±0.040^Bc^ | 0.107±0.044^Bb^ | 38.18 |

Note: C, (+)-catechin; EC, (-)-epicatechin; EGC, (-)-epigallocatechin; ECG, (-)-epicatechin gallate; GCG, (-)-gallocatechin gallate; EGCG, (-)-epigallocatechin gallate; CG, (-)-catechin gallate; TGG, 1,3,6-tri-*O-*galloyl-β-D-glucose.

Different uppercase and lowercase letters in superscript (A, B and C, *P* < 0.001; a,b, c and d, *P* < 0.05) in a same row indicate levels of statically significant difference determined by one-way ANOVA using Duncan`s multiple range test. F-value was determined by ANOVA through SPSS 20.0 software.

**Table S2.** Differences of *in vitro* antioxidant capacity in ripened Pu-erh tea (RiPT) among four series.

| Index | A (n=5×3×2) | B (n=5×3×2) | C (n=5×3×2) | D (n=5×3×2) | F-value |
| --- | --- | --- | --- | --- | --- |
| T-AOC (μmol Trolox/g) | 215.7±88.8^Bb^ | 201.2±88.1^Bb^ | 365.7±79.8^Aa^ | 205.7±40.1^Bb^ | 31.93 |
| DRSA(mg Trolox/g) | 36.30±10.54^Bb^ | 35.41±11.14^Bb^ | 129.1±64.8^Aa^ | 36.29±5.91^Bb^ | 58.12 |
| ARSA(mg Trolox/g) | 11.22±2.87^Bb^ | 12.20±3.09^Bb^ | 27.77±10.92^Aa^ | 13.31±1.87^Bb^ | 52.08 |
| HRSA(%) | 32.66±5.02^Bb^ | 32.59±3.75^Bb^ | 36.95±2.38^Aa^ | 35.84±3.13^ABa^ | 10.85 |
| SARSA(%) | 46.57±9.38^BCb^ | 52.21±3.42^Aa^ | 44.09±4.48^Cb^ | 50.02±5.07^ABa^ | 10.70 |

Note: T-AOC, total antioxidant capacity; DRSA, DPPH radical scavenging ability; ARSA, ABTS radical scavenging ability; HRSA, hydroxyl radical scavenging ability; SARSA, superoxide anion radical scavenging ability.

Different uppercase and lowercase letters in superscript (A, B and C, *P* < 0.001; a,b, c and d, *P* < 0.05) in a same row indicate levels of statically significant difference determined by one-way ANOVA using Duncan`s multiple range test. F-value was determined by ANOVA through SPSS 20.0 software.

**Table S3**. Inhibitory activities of various concentrations of ripened Pu-erh tea extract (RiPTE) on *α*-amylase and *α*-glucosidase, and their differences among four series.

| Index | Concentration | A (n=5×3×2) | B (n=5×3×2) | C (n=5×3×2) | D (n=5×3×2) | F-value |
| --- | --- | --- | --- | --- | --- | --- |
| Inhibitory rate (%) on α-amylase | 10 mg/mL | 23.65±2.87^Aa^ | 24.09±3.52^Aa^ | 24.81±4.71^Aa^ | 23.37±3.34^Aa^ | 0.879 |
|  | 20 mg/mL | 37.63±5.42^Ab^ | 40.10±6.73^Aab^ | 41.49±6.02^Aa^ | 39.32±7.11^Aab^ | 1.927 |
|  | 30 mg/mL | 47.38±6.78^Ab^ | 52.23±7.74^Aa^ | 53.29±7.41^Aa^ | 53.13±10.20^Aa^ | 3.530 |
|  | 40 mg/mL | 55.06±4.86^Bb^ | 61.95±12.77^ABa^ | 67.39±11.63^Aa^ | 61.92±11.86^ABa^ | 6.609 |
|  | 50 mg/mL | 70.54±6.20^Ab^ | 74.27±13.31^Aab^ | 79.37±9.88^Aa^ | 75.62±10.27^Aab^ | 3.816 |
| Inhibitory rate (%) on α-glucosidase | 50 µg/mL | 9.95±1.72^Aa^ | 8.53±0.95^Bb^ | 8.63±1.46^Bb^ | 9.14±1.18^ABb^ | 6.823 |
|  | 100 µg/mL | 23.31±3.86^Aa^ | 19.56±2.67^Bb^ | 19.07±3.16^Bb^ | 19.84±1.98^Bb^ | 12.507 |
|  | 200 µg/mL | 44.44±5.30^Aa^ | 38.08±3.20^Bb^ | 37.88±6.10^Bb^ | 40.28±4.03^ABb^ | 12.161 |
|  | 400 µg/mL | 66.43±5.65^Aa^ | 61.00±6.78^ABb^ | 57.47±7.83^Bb^ | 60.47±6.43^ABb^ | 9.258 |
|  | 500 µg/mL | 81.99±4.82^Aa^ | 75.04±6.84^Bc^ | 76.52±9.72^ABbc^ | 80.70±6.43^ABab^ | 6.533 |

Different uppercase and lowercase letters in superscript (A, B and C, *P* < 0.001; a,b, c and d, *P* < 0.05) in a same row indicate levels of statically significant difference determined by one-way ANOVA using Duncan`s multiple range test. F-value was determined by ANOVA through SPSS 20.0 software.

**Table S4.** Differences of *in vitro* antioxidant capacity in ripened Pu-erh tea (RiPT) among five grades.

| Index | G1 (n=4×3×2) | G3 (n=4×3×2) | G5 (n=4×3×2) | G7 (n=4×3×2) | G9 (n=4×3×2) | F-value |
| --- | --- | --- | --- | --- | --- | --- |
| T-AOC (μmol Trolox/g) | 281.1±102.6^Aa^ | 267.7±103.7^ABa^ | 270.3±101.7^ABa^ | 237.6±120.0^ABa^ | 178.7±34.5^Bb^ | 4.37 |
| DRSA(mg Trolox/g) | 46.65±10.08^Aab^ | 70.63±62.81^Aa^ | 75.13±62.73^Aa^ | 68.90±69.25^Aa^ | 35.08±8.74^Ab^ | 2.84 |
| ARSA(mg Trolox/g) | 14.06±2.81^Aab^ | 17.65±10.24^Aa^ | 18.79±10.65^Aa^ | 18.23±12.15^Aa^ | 11.91±2.20^Ab^ | 2.84 |
| HRSA(%) | 35.15±3.39^ABab^ | 34.55±3.94^ABab^ | 36.63±4.04^Aa^ | 34.08±4.67^ABbc^ | 32.16±3.46^Bc^ | 4.12 |
| SARSA(%) | 43.34±7.15^Bc^ | 46.20±4.27^ABbc^ | 49.28±5.81^ABab^ | 50.41±8.41^Aa^ | 51.89±3.34^Aa^ | 7.66 |
| α-amylase IR(%) | 88.47±7.54^Aa^ | 78.00±8.81^Bb^ | 71.83±4.94^BCc^ | 71.34±7.64^BCc^ | 64.71±5.35^Cd^ | 38.77 |
| α-glucosidase IR(%) | 86.44±4.77^Aa^ | 80.26±5.88^Bb^ | 77.89±5.17^Bb^ | 76.94±5.26^BCb^ | 71.36±7.92^Cc^ | 20.56 |

Note: T-AOC, total antioxidant capacity; DRSA, DPPH radical scavenging ability; ARSA, ABTS radical scavenging ability; HRSA, hydroxyl radical scavenging ability; SARSA, superoxide anion radical scavenging ability; IR, inhibitory rate.

Different uppercase and lowercase letters in superscript (A, B and C, *P* < 0.001; a,b, c and d, *P* < 0.05) in a same row indicate levels of statically significant difference determined by one-way ANOVA using Duncan`s multiple range test. F-value was determined by ANOVA through SPSS 20.0 software.


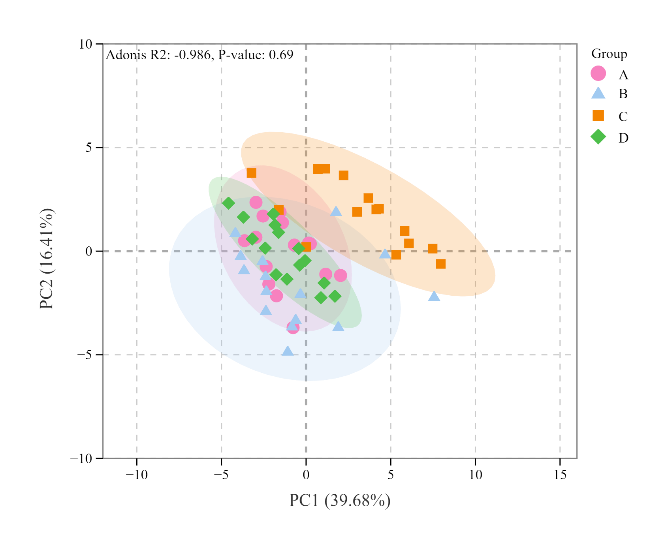

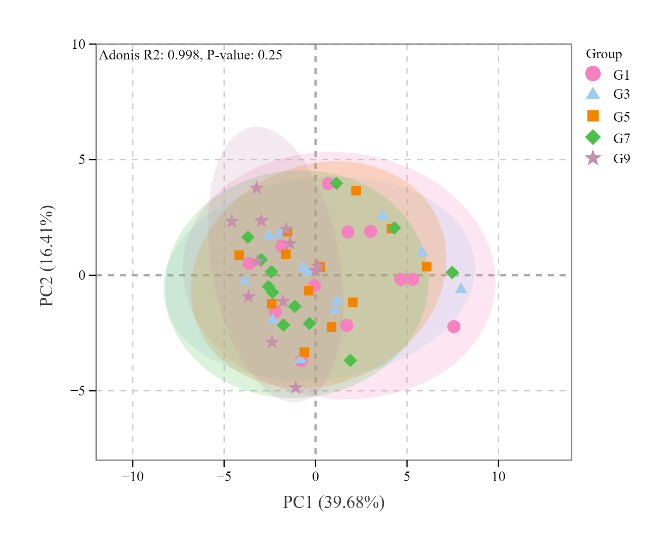


**Fig. S1.** Principal component analysis (PCA) of 20 ripened Pu-erh tea (RiPT) samples for classification of series (a) and grade (b), respectively.
